# Interferon-γ and Proliferation Responses to *Salmonella enterica* Serotype Typhi Proteins in Patients with *S*. Typhi Bacteremia in Dhaka, Bangladesh

**DOI:** 10.1371/journal.pntd.0001193

**Published:** 2011-06-07

**Authors:** Alaullah Sheikh, Farhana Khanam, Md. Abu Sayeed, Taibur Rahman, Marcin Pacek, Yanhui Hu, Andrea Rollins, Md. Saruar Bhuiyan, Sean Rollins, Anuj Kalsy, Mohammad Arifuzzaman, Daniel T. Leung, David A. Sarracino, Bryan Krastins, Richelle C. Charles, Regina C. LaRocque, Alejandro Cravioto, Stephen B. Calderwood, W. Abdullah Brooks, Jason B. Harris, Joshua LaBaer, Firdausi Qadri, Edward T. Ryan

**Affiliations:** 1 International Centre for Diarrhoeal Disease Research, Bangladesh (ICDDR,B), Dhaka, Bangladesh; 2 Division of Infectious Diseases, Massachusetts General Hospital, Boston, Massachusetts, United States of America; 3 Harvard Institute of Proteomics, Cambridge, Massachusetts, United States of America; 4 Department of Medicine, Harvard Medical School, Boston, Massachusetts, United States of America; 5 Thermo Fisher Scientific, Cambridge, Massachusetts, United States of America; 6 Department of Microbiology and Molecular Genetics, Harvard Medical School, Boston, Massachusetts, United States of America; 7 Arizona State University, Tempe, Arizona, United States of America; 8 Department of Immunology and Infectious Diseases, Harvard School of Public Health, Boston, Massachusetts, United States of America; Mahidol University, Thailand

## Abstract

**Background:**

*Salmonella enterica* serotype Typhi is a human-restricted intracellular pathogen and the cause of typhoid fever. Cellular immune responses are required to control and clear *Salmonella* infection. Despite this, there are limited data on cellular immune responses in humans infected with wild type *S.* Typhi.

**Methodology/Principal Findings:**

For this work, we used an automated approach to purify a subset of *S.* Typhi proteins identified in previous antibody-based immuno-affinity screens and antigens known to be expressed in vivo, including StaF-putative fimbrial protein-STY0202, StbB-fimbrial chaperone-STY0372, CsgF-involved in curli production-STY1177, CsgD- putative regulatory protein-STY1179, OppA-periplasmic oligopeptide binding protein precursor-STY1304, PagC-outer membrane invasion protein-STY1878, and conserved hypothetical protein-STY2195; we also generated and analyzed a crude membrane preparation of *S*. Typhi (MP). In comparison to samples collected from uninfected Bangladeshi and North American participants, we detected significant interferon-γ responses in PBMCs stimulated with MP, StaF, StbB, CsgF, CsgD, OppA, STY2195, and PagC in patients bacteremic with *S*. Typhi in Bangladesh. The majority of interferon-γ expressing T cells were CD4 cells, although CD8 responses also occurred. We also assessed cellular proliferation responses in bacteremic patients, and confirmed increased responses in infected individuals to MP, StaF, STY2195, and PagC in convalescent compared to acute phase samples and compared to controls. StaF is a fimbrial protein homologous to *E. coli* YadK, and contains a Pfam motif thought to be involved in cellular adhesion. PagC is expressed in vivo under the control of the virulence-associated PhoP-regulon required for intra-macrophage survival of *Salmonella*. STY2195 is a conserved hypothetical protein of unknown function.

**Conclusion/Significance:**

This is the first analysis of cellular immune responses to purified *S*. Typhi antigens in patients with typhoid fever. These results indicate that patients generate significant CD4 and CD8 interferon-γ responses to specific *S*. Typhi antigens during typhoid fever, and that these responses are elevated at the time of clinical presentation. These observations suggest that an interferon-γ based detection system could be used to diagnose individuals with typhoid fever during the acute stage of illness.

## Author Summary

*Salmonella enterica* serotype Typhi infection is a significant global public health problem and the cause of typhoid fever. *Salmonella* are intracellular pathogens, and cellular immune responses are required to control and clear *Salmonella* infections. Despite this, there are limited data on cellular immune responses during wild type *S*. Typhi infection in humans. Here we report the assessment of cellular immune responses in humans with *S*. Typhi bacteremia through a screening approach that permitted us to evaluate interferon-γ and proliferation responses to a number of *S*. Typhi antigens. We detected significant interferon-γ CD4 and CD8 responses, as well as proliferative responses, to a number of recombinantly purified *S*. Typhi proteins as well as membrane preparation in infected patients. Antigen-specific interferon-γ responses were present at the time of clinical presentation in patients and absent in healthy controls. These observations could assist in the development of interferon-γ-based diagnostic assays for typhoid fever.

## Introduction

*Salmonella enterica* serotype Typhi is a human-restricted intracellular pathogen and the cause of typhoid fever. It is estimated that over 20 million cases of *S.* Typhi infection occur each year, resulting in approximately 200,000 deaths per year globally [1]. Current typhoid vaccines provide 50–75% protection for 2–5 years [2]. Mediators of protective immunity against typhoid are incompletely understood. *S.* Typhi is an invasive enteropathogen that, following ingestion, transits through intestinal epithelial cells, is taken up by professional phagocytic cells, survives within macrophages, and systemically circulates [3], [4], [5], [6]. Antibody responses to lipopolysaccharide (LPS), flagellin, Vi capsular polysaccharide, and crude whole cell preparations have been documented, and antibody responses are the basis of the Widal serologic diagnostic assay for typhoid fever [7], [8], [9], [10], [11]. However, with the exception of antibody responses against the *S.* Typhi capsule (Vi antigen) [12], antibody responses may play a limited role in mediating protective immunity during typhoid fever.

*S*. Typhi is an intracellular pathogen, and cellular immune responses are required to control and clear *S*. Typhi infections [10], [13], [14], [15]. Unfortunately, there are limited data on antigen-specific cellular responses during human wild type *S*. Typhi infection. What is known is largely derived from analyses of cellular responses in mice infected with *S.* Typhimurium [16], [17], [18]; however, *S.* Typhimurium does not cause a typhoidal illness in humans, and *S.* Typhi and *S.* Typhimurium differ significantly at the genomic level [17], [19], [20]. Direct analysis of cellular responses during *S*. Typhi infection in humans either pre-dates modern immunologic techniques [21] or involves characterizing immune responses in recipients of live attenuated oral typhoid vaccines [22], [23]. These analyses have shown that CD4^+^ and CD8^+^ T cells are critical to the development of protective immunity to *Salmonella*, and control of *Salmonella* infection involves prominent expression of interferon-γ by both CD4 and CD8 cells [24], [25], [26]. To date, however, there is less information on the cellular responses in humans during wild type infection, especially to purified *S*. Typhi antigens.

To address this, we used a modification of an automated approach to purify a subset of *S*. Typhi proteins for use in immunologic assays [27]. We selected antigens for evaluation based on our previous application of a high throughput immuno-affinity screen, In Vivo Induced Antigen Technology (IVIAT), to *S*. Typhi [28]. Here we describe purification of a subset of these proteins, and evaluation of interferon-γ and cellular proliferation responses to these antigens in humans with *S*. Typhi bacteremia in Bangladesh. We also assessed responses to a crude membrane preparation of *S*. Typhi.

## Materials and Methods

### Generation of expression clones for antigen production

We selected 58 *S*. Typhi proteins contained within operons identified during our previous application of IVIAT to *S*. Typhi [28]. IVIAT identifies proteins expressed in vivo during human infection and that generate an antibody response [28]. We obtained pDONR221 Gateway Based entry clones of the *S*. Typhi CT18 genes corresponding to selected proteins from the NIAID-sponsored Pathogen Functional Genomic Resource Center, J. Craig Venter Institute (JCVI, formerly The Institute for Genomic Research). We used LR clonase II enzyme reactions (Invitrogen, Carlsbad, CA) as per the manufacturer's instructions to move inserts into pDEST17 (Invitrogen, Carlsbad, CA) to generate a fusion containing an amino terminal 6× histidine (HIS) tag. We transformed DH5alpha-T1R competent cells with LR reactions and selected for ampicillin resistance. We confirmed insert presence by restriction digestion and PCR analysis and transformed purified plasmids into *E. coli* protein expression strain BL21 star (DE3) pLysS (Invitrogen).

### Protein expression

We grew transformants harboring recombinant plasmids at 37°C as 1.5 ml cultures in 96-well blocks (Marsh Biomedical Products) to an OD_600_ of 0.6–0.8. We induced cultures with 1 mM isopropyl β-D-1-thiogalactopyranoside (IPTG) on a 96-well plate shaker (Multitron) (×900 rpm). After 3 hours at 37°C, we harvested cells at 4°C and stored preparations at −80°C for further use. We also induced BL21 star (DE3) pLysS containing pDEST17 but lacking an *S*. Typhi insert. This construct produced a truncated HIS-tagged protein MSYYHHHHHHLESTSLYKKAERERKMI that we recovered and used as a control protein in immunological assays.

### Automated 96-well protein purification

We performed protein purifications in 96-well plates using a BiomekFx (Beckman Coulter) robotic liquid handler as previously described [27]. For this 6xHIS denaturing affinity purification, we thawed cell pellets at room temperature for 15 min, lysed them in the presence of protease inhibitors in 115 µl lysis buffer I (100 mM NaH_2_PO_4,_ 10 mM Tris, pH 8.0), robotically resuspended product in a 96-well block and agitated at 900 rpm for 10 min (5 min in the clockwise direction and 5 min in the counterclockwise direction). We then added 10 µl of DNase mix (10 mg/ml DNase; Sigma Aldrich in 900 mM MgCl_2_, 100 mM MnCl_2_) to the lysate and agitated the preparation at 900 rpm for 10 min. Next, we added 115 µl of lysis buffer II (100 mM NaH_2_PO_4,_ 10 mM Tris, 6 M guanidine hydrochloride, 10 mM, 2-mercaptoethanol, pH 8.0) to create denaturing conditions. We then allowed these cell lysates to bind to 30 µl of MagneHIS beads (Promega) with shaking at 900 rpm for 20 min (10 min clockwise, 10 min counterclockwise), and separated beads using a magnabot (24-pin magnet; Promega). The robotic liquid handler then washed the MagneHIS beads with bound protein three times with wash buffer (100 mM NaH_2_PO_4,_ 10 mM Tris, 8 M urea). We prevented bead adherence to the walls during washing by shaking the samples at 900 rpm for 2.5 min clockwise and then 2.5 min counterclockwise. We then washed the beads with bound protein using 100 µl of distilled water, and added 50 µl distilled water to make the final suspensions for analysis. We repeated this extraction cycle six times.

### Automated 96-well protein analysis

We analyzed proteins in a 96-well format using a capillary-based instrument, the LabChip90 (Caliper Sciences). We automated a system that resuspended 3 µl of protein sample in 7 µl analysis buffer (Caliper Sciences), heated these to 96°C for 5 min., cooled them to room temperature, and briefly centrifuged to collect the sample. We added distilled water (35 µl) to each sample prior to analysis. We primed the analysis chip (Caliper Sciences) according to the manufacturer’s instructions. The automated protein analysis generated three different forms of output: a chromatogram that showed migration time; a virtual gel that mimicked a Coomassie stained gel; and a results table that included the estimated size, quality, and quantity of each peak. The LabChip90 analyzed 96 proteins at a time with analysis time of 40 seconds per sample. We parsed the output results and imported them into the Harvard Institute of Proteomics protein database. We assessed for presence of contaminating *E. coli* LPS using a HEK-Blue LPS Detection kit (InvivoGen, San Diego, CA).

### Production and mass spectrometric analysis of *S*. Typhi crude membrane preparation

We prepared *S*. Typhi membrane preparation as previously described [29], [30]. Briefly, we cultured *S*. Typhi Ty21a on sheep blood agar plates and harvested in Tris buffer (10 mM Tris, pH 8.0, 5 mM MgCl_2_). We sonicated the mixture, and centrifuged at 1400× g for 10 minutes and transferred the supernatant to fresh tubes, centrifuging at 14900× g for 30 minutes. We suspended the pellet in 10 ml Tris buffer, and determined the protein content by the BioRad Protein Assay per the manufacturer's instructions.

We performed mass spectrometric analysis of the *S*. Typhi membrane preparation as previously described using a LTQ-Orbitrap XL (Thermo Fisher Scientific) instrument [19], [31]. We identified peptides using SEQUEST (Thermo Fisher Scientific) through Bioworks Browser, version 3.3.1 SR1. MS/MS data were obtained using 10 ppm mass accuracy on precursor m/z and a 0.5 Da window on fragment ions. Fully enzymatic tryptic searches with up to three missed cleavage sites were allowed. Oxidized methionines were searched as a variable modification and alkylated cysteines were searched as a fixed modification. *Salmonella* databases for CT18 were downloaded from EMBL-EBI and supplemented with common contaminants. We employed a reverse database strategy [32] using concatenating reversed protein sequences for each database entry in SEQUEST. We filtered peptides for each charge state to a false discovery rate (FDR) of 1%, and then grouped peptides into proteins using Occam’s razor logic. A full listing of proteins identified in mass spectrometric analysis of *Salmonella* Typhi membrane preparation is available in the supplemental material (Table S1).

### Collection of specimens from study subjects

Individuals (1–59 years of age) with fever of 3–7 days duration (≥39°C) having clinical symptoms and signs suggestive of typhoid fever and lacking an alternate diagnosis who presented to the Kamalapur field site of the International Centre for Diarrhoeal Disease Research, Bangladesh (ICDDR,B) Dhaka hospital were eligible for enrollment. We collected venous blood (for children <5 years of age, 3 ml of blood; for older individuals, 5 ml of blood) for culture (n = 69). We used the BacT/Alert automated system and identified *S*. Typhi organisms using standard biochemical methods and by reaction with *Salmonella*-specific antisera [30], [33]. Following informed consent from patients or guardians in the case of children, we collected an additional 5 ml of blood from bacteremic individuals within 72 hours of the patient presenting for medical care, and a follow-up sample 21–28 days later (n = 16; ages 2–22 years). All patients with 3 days or longer of fever were treated initially with amoxicillin or cefixime at the discretion of the attending physician until scheduled follow-up 48–72 hours later, or sooner as clinically indicated. Individuals with documented *S*. Typhi bacteremia were continued on amoxicillin if they showed signs of improvement and their blood isolates showed sensitivity to first line treatment; or were switched to parenteral ceftriaxone or oral ciprofloxacin, if their isolates were not sensitive and/or they failed to improve by 72 hours; therapy was continued for up to 14 days, or up to 7 days beyond defervescence, whichever occurred first. All patients recovered. We also collected 5 ml of blood from North American volunteers (n = 3) without a history of international travel who had never received typhoid vaccination and who did not have previous known *Salmonella* infection, and we collected 5 ml of blood from healthy Bangladeshi volunteers (n = 4) who did not have illness, fever or diarrhea in the preceding three months [34]. Studies were approved by the Institutional Review Boards of the ICDDR,B and Massachusetts General Hospital.

### PBMC isolation

We diluted heparinized blood in phosphate buffered saline (PBS; 10 mM, pH 7.2) and isolated peripheral blood mononuclear cell (PBMC) by gradient centrifugation on Ficoll-Isopaque (Pharmacia, Uppsala, Sweden). We re-suspended isolated PBMCs to a concentration of 1×10^6^ cells/ml in RPMI complete medium RPMI-1640 (Gibco, Gaithersburg, Md) with 10% heat-inactivated fetal bovine serum (Hyclone-Thermo Scientific, Waltham, MA, USA), 100 units/ml penicillin, 100 µg/ml streptomycin, 100 mM pyruvate, and 200 mM L-glutamine (Gibco) [35].

### Interferon gamma ELISPOT assay

We used PBMCs to measure human interferon-γ expression using an ELISPOT format with MabTech antibodies, according to the manufacturers’ instructions (Mabtech Inc, Cincinati, OH, USA). In brief, we coated 96-well nitrocellulose plates (Multiscreen HTS, Millipore) with 100 µl of 15 µg/ml human monoclonal anti-interferon-γ antibody (1-D1K) overnight at 4°C. Following washing the plates and subsequent blocking with 10% FBS for 2 h at room temperature, we added PBMCs from individual patients or controls at a concentration of 2×10^5^ per well for each experimental condition. We added individual *S*. Typhi antigens or control protein to wells at a concentration of 140 ng/well of total preparation for each purified antigen (in 200 µl culture, final concentration 0.7 µg/ml). In separate wells, we also added *S*. Typhi membrane preparation at a final concentration of 10 µg/ml in 200 µl culture, phytohaemagglutinin (PHA; Murex Diagnostics Ltd, Temple Hill, UK) at a final concentration of 2.5 µg/ml in 200 µl culture, and keyhole limpet hemocyanin (KLH). We included additional control wells with media but lacking antigen. Following incubation of plates at 37°C in 5% CO_2_ for 20 hours, we washed plates, added biotinylated monoclonal anti-interferon-γ antibody (7-B6-1-biotin; 1∶500 dilution), incubated plates at room temperature for an additional 2 hours, washed them, added streptavidin-HRP (1∶500 dilution), and re-incubated for 1 hour at room temperature. We developed plates with aminoethylcarbazol plus H_2_O_2_, and counted interferon-γ secreting cells using a stereomicroscope. We subtracted results for wells containing media only and expressed results as the number of spots/10^6^ PBMC in each experimental condition [36].

### Intracellular cytokine staining

To characterize the interferon-gamma T cell response further, we resuspended PBMCs at a concentration of 1×10^6^ cells/mL in RPMI medium (Gibco, Carlsbad, CA) and supplemented with 10% fetal calf serum (FCS, Gibco). We cultured PBMCs in U-bottom tissue culture plates (Nunc, Denmark) in the presence of *Salmonella* membrane preparation (MP; 10 µg/ml), StaF (7 µg/ml), PagC (7 µg/ml), KLH (2.5 µg/ml as a negative control) or PMA (5.0 ng/ml as a positive control; Phorbol 12-myristate 13-acetate) with ionomycin (1.0 µg/ml). Samples containing only unstimulated cells were included to assess *in vivo* stimulation. We used 1.0 µg/ml of anti-CD28 (clone 28.2; BD Pharmingen) and anti-CD49d (clone 9F10; BD Pharmingen) for co-stimulation. We incubated PBMCs and antigens for 2 hours at 37° C in 5% CO_2_. After 2 hours, we added 10 µg/mL of brefeldin A (BFA, Sigma) and continued incubating the plates for an additional 4 hours [37]. Following stimulation, we washed cells with PBS and 2% FCS. We then stained cells for 30 min at 4°C with the following surface monoclonal antibodies: anti-CD3-APC, anti-CD4–perCP, and anti-CD8-FITC (Becton Dickinson, San Jose, USA). Following surface staining, we washed the cells and incubated the preparations with FACS Lysing Solution (BD Bioscience) for 10 minutes, and then re-washed and permeabilized the preparations with FACS permeabilizing solution (BD Bioscience) for 10 min at room temperature. We washed the permeabilized cells and stained them for 30 min at 4°C with fluorochrome-conjugated anti-IFN-γ-PE (BD Bioscience). Following staining, we re-washed the cells, and fixed them in formaldehyde before performing flow cytometry using a FACS Calibur (BD, San Jose, CA) [37]. We identified the lymphocyte population on forward versus side scatter plot, then gated CD3^+^CD4^+^ and CD3^+^CD8^+^ subpopulations, and identified CD4^+^IFN-γ^+^ and CD8^+^IFN-γ^+^ subpopulations. We subtracted unstimulated responses, and expressed results as interferon-γ^+^ T cells per 10×6 PBMC.

### T-cell proliferation assay

To evaluate proliferative responses to antigens, we cultured PBMCs (10^5^ cell per well) in DMEM/F12 medium (Gibco, GlutaMAX) supplemented with 1% gentamicin and 5% human AB+ serum in triplicate wells in round-bottomed 96-well plates. We added *S*. Typhi antigens and controls to wells at the same concentrations used in the interferon-γ ELISPOT assay and with a final culture volume of 200 µl. We incubated plates at 37°C in 5% CO_2_ for 5 days. After 48 h incubation, we replaced 100 µl of the medium per well with fresh medium. After 5 days of incubation, we added ^3^H-thymidine (1 µCi) to each well under sterile conditions, incubated plates for an additional 8 hours, harvested cells in Bray’s scintillation fluid (Ultimagold, PerkinElmer, Boston, MA) using a cell harvester (Skatron instruments, Norway), and assessed [^3^H] thymidine incorporation using a liquid scintillation β-counter (Beckman LS6500 multipurpose scintillation counter, USA) as previously described [22], [38]. We expressed results as counts per minute (cpm), and calculated stimulation indices for each antigen according to the formula: net cpm with antigen /net cpm without antigen (media alone) for each individual on each day (day 5 and day 20) [39].

### Statistical analysis

We used Prism4 (version 4.03, GraphPad Software, Inc.) for data management, analysis and graphical presentation. We used unpaired T tests to compare differences between groups, and paired T tests to evaluate differences between study days within groups.

## Results

### Automated production of *S*. Typhi proteins

We estimated that we required at least 20 µg of a specific protein for use in our planned immunological assays. Our six production runs resulted in the production of 20 µg or more for 25 of our selected 58 proteins; nine of these samples had purity by LC90 Caliper analysis of >90%, and 17 had purity greater than >80%. Purity was defined as the quantity of protein matching the molecular size of the desired product. The LPS contamination of all preparations was found to be less than the level of detection of our assay kit (<300 fg/µl). Of these 17 proteins with sufficient quantity and purity, we selected 7 proteins for our initial analysis (Table 1) representing a range of cellular location and function, including a number involved in fimbrial attachment or adhesion such as StaF (putative fimbrial protein encoded by STY0202), StbB (fimbrial chaperone encoded by STY0372), CsgF (involved in curli production encoded by STY1177), and CsgD (a putative regulatory protein encoded by STY1179), as well as OppA (a periplasmic oligopeptide binding protein precursor involved in peptide transport encoded by STY1304), a conserved hypothetical protein encoded by STY2195, and PagC, an outer membrane protein encoded by STY1878 whose expression is regulated by the PhoP regulon involved in intra-macrophage survival [19], [28].

**Table 1:** *S*. Typhi protein preparations used in this study.

STY number	Annotated name	Protein
**STY0202**	Putative fimbrial protein	**StaF**
**STY0372**	Fimbrial chaperone protein	**StbB**
**STY1177**	Assembly/transport component in curli production	**CsgF**
**STY1179**	Putative regulatory protein	**CsgD**
**STY1304**	Periplasmic oligopeptide-binding protein precursor	**OppA**
**STY1878**	Outer membrane invasion protein	**PagC**
**STY2195**	Conserved hypothetical protein	
**Membrane preparation**	Crude membrane preparation containing at least 934 *S*. Typhi proteins (see Tables 2 and 3)	**MP**

### Mass spectrometric analysis of the *S*. Typhi membrane preparation

Our mass spectrometric analysis of *S*. Typhi membrane preparation identified 934 *S*. Typhi proteins (636 with three or more spectral counts), including many involved in energy metabolism, protein synthesis and fate, cell envelope or peptidoglycan synthesis or maintenance, cellular processes, proteins involved in transport, proteins involved in regulatory functions, and proteins involved in virulence and pathogenesis (Table 2 and 3 and Table S1). We also identified two of our 7 selected proteins (OppA and PagC) in the *S*. Typhi membrane preparation.

**Table 2:** Functional categories of proteins detected in *S*. Typhi membrane preparation.

Classification	Number of proteins
Energy metabolism	134
Unknown function or unclassified	105
Cell envelope	90
Hypothetical proteins	83
Protein synthesis	77
Transport and binding proteins	74
Protein fate	65
Pathogenesis/virulence/cellular processes	59
Central intermediary metabolism	42
Regulatory functions	39
DNA metabolism	38
Amino acid biosynthesis	30
Biosynthesis of cofactors, prosthetic groups, and carriers	28
Purines, pyrimidines, nucleosides, and nucleotides	25
Transcription	19
Fatty acid and phospholipid metabolism	18
Mobile and extrachromosomal element functions	4
Viral functions	4
**Total**	**934**

**Table 3:** Proteins represented in the *S.* Typhi membrane preparations and selected *S*. Typhi antigens.

Accession	Entry name	Protein name	Peptide Hits	Rank*
**Most abundant proteins**				
P0A1H6	EFTU_SALTI	Elongation factor Tu (EF-Tu)	380	1
Q8Z7S0	OMPA_SALTI	Outer membrane protein A (OmpA)	342	2
Q8Z8C8	Q8Z8C8_SALTI	Succinate dehydrogenase flavoprotein subunit	298	3
Q8Z1T9	LAMB_SALTI	Maltoporin (maltose-inducible porin)	250	4
Q8XGX4	ATPB_SALTI	ATP synthase subunit beta	201	5
Q8XG95	ATPA_SALTI	ATP synthase subunit alpha	185	6
P0A264	OMPC_SALTI	Outer membrane protein C (porin ompC)	170	7
Q8Z9F0	Q8Z9F0_SALTI	Pyruvate dehydrogenase E1 component	135	8
Q8Z4L6	PUR4_SALTI	Phosphoribosylformylglycinamidine synthase	132	9
P0AA29	THIO_SALTI	Thioredoxin-1 (Trx-1)	125	10
Q8Z9E6	Q8Z9E6_SALTI	Aconitate hydratase 2	117	11
Q8Z937	FADE_SALTI	Acyl-coenzyme A dehydrogenase	116	12
P0A1D4	CH60_SALTI	60 kDa chaperonin pProtein Cpn60) (GroEL protein)	105	13
Q8Z9A3	YAET_SALTI	Outer membrane protein assembly factor yaeT	102	14
Q8XH17	Q8XH17_SALTI	Outer membrane protein x	98	15
Q8Z858	Q8Z858_SALTI	D-alanyl-D-alanine carboxypeptidase (penicillin-binding protein 6)	91	16
Q8Z6J0	Q8Z6J0_SALTI	Phosphoenolpyruvate synthase	88	17
Q8XFH6	Q8XFH6_SALTI	Peptidoglycan-associated lipoprotein	86	18
Q8Z8C6	Q8Z8C6_SALTI	2-oxoglutarate dehydrogenase E1 component	78	19
Q8Z7D2	Q8Z7D2_SALTI	Aconitate hydratase 1 (Aconitate hydratase 1 (citrate hydro-lyase 1))	75	20
**Selected virulence proteins**				
Q8Z7H3	PHOQ_SALTI	Virulence sensor histidine kinase (PhoQ)	70	29
Q8Z8L8	Q8Z8L8_SALTI	Ferrienterobactin receptor (FepA)	38	103
Q8Z7H2	PHOP_SALTI	Virulence transcriptional regulatory protein (PhoP)	37	104
Q8Z1P4	Q8Z1P4_SALTI	Two-component response regulator (PmrA)	4	487
P61091	SLYA_SALTI	Transcriptional regulator (SlyA)	4	543
Q8Z6B2	Q8Z6B2_SALTI	Outer membrane invasion protein (PagC)	4	544
Q8Z3Y7	Q8Z3Y7_SALTI	Putative uncharacterized protein associated with virulence (STY3182)	2	749
Q8Z727	HLYE_SALTI	Hemolysin E (Cytotoxin ClyA)	1	859
***S*** **. Typhi proteins individually purified and also detected in ** ***S*** **. Typhi membrane preparation**				
Q8Z7F0	Q8Z7F0_SALTI	Periplasmic oligopeptide-binding protein (OppA)	15	225
Q8Z6B2	Q8Z6B2_SALTI	Outer membrane invasion protein (PagC)	4	544

*Rank order of abundance.

### Interferon-γ ELISPOT responses and T cell characterization

**Figure 1:**
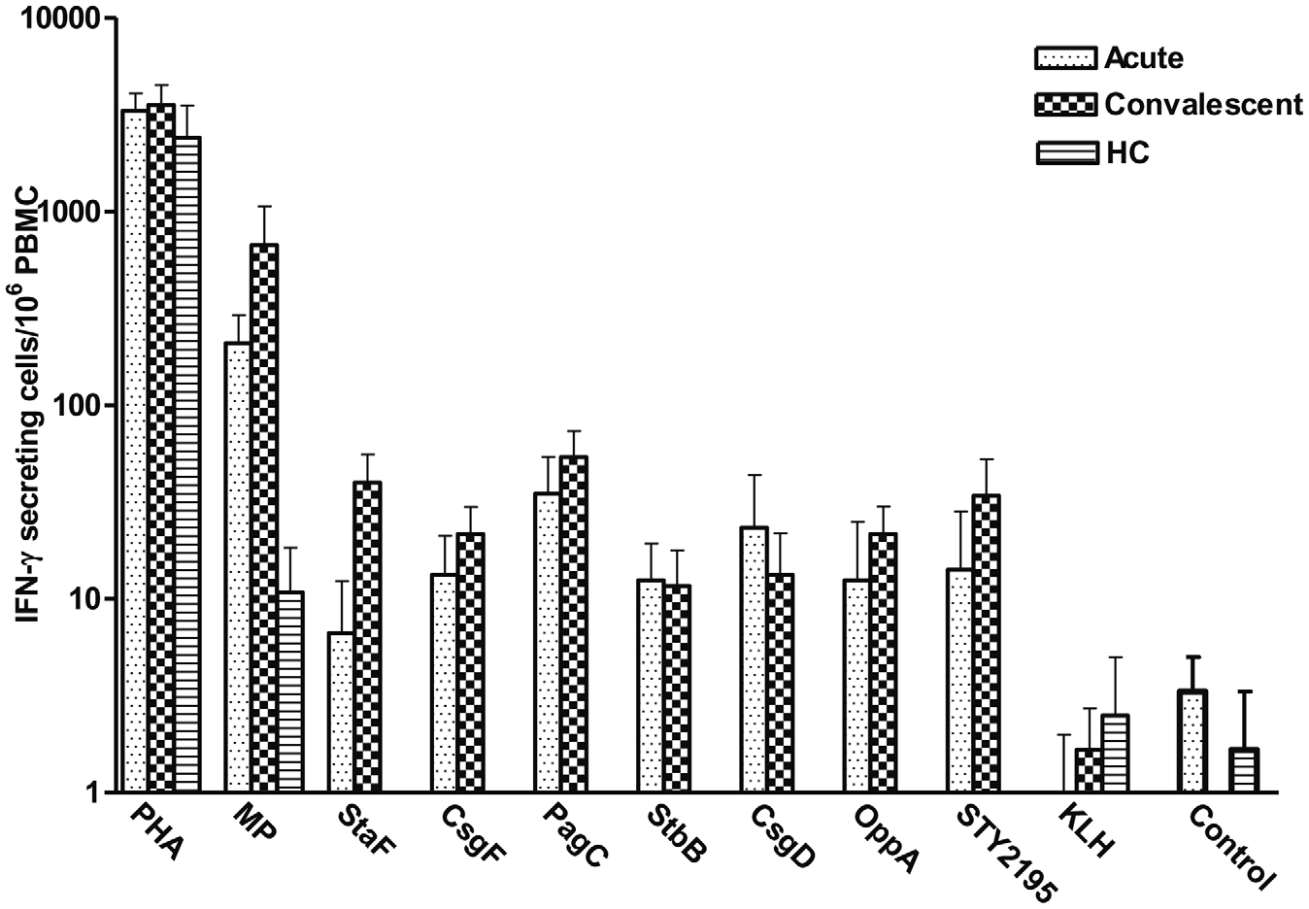
Interferon-γ ELISPOT responses to *S*. Typhi antigens including StaF (STY0202), CsgF (STY1177), PagC (STY1878), StbB (STY0372), CsgD (STY1179), OppA (STY1304), conserved hypothetical protein encoded by STY2195, *S*. Typhi membrane preparation (MP), control protein, and phytohaemagglutinin (PHA) and keyhole limpet hemocyanin (KLH) during acute and convalescent stage illness in *S*. Typhi bacteremic patients and in healthy controls (HC). Mean and standard error of the mean represented.

**Figure 2:**
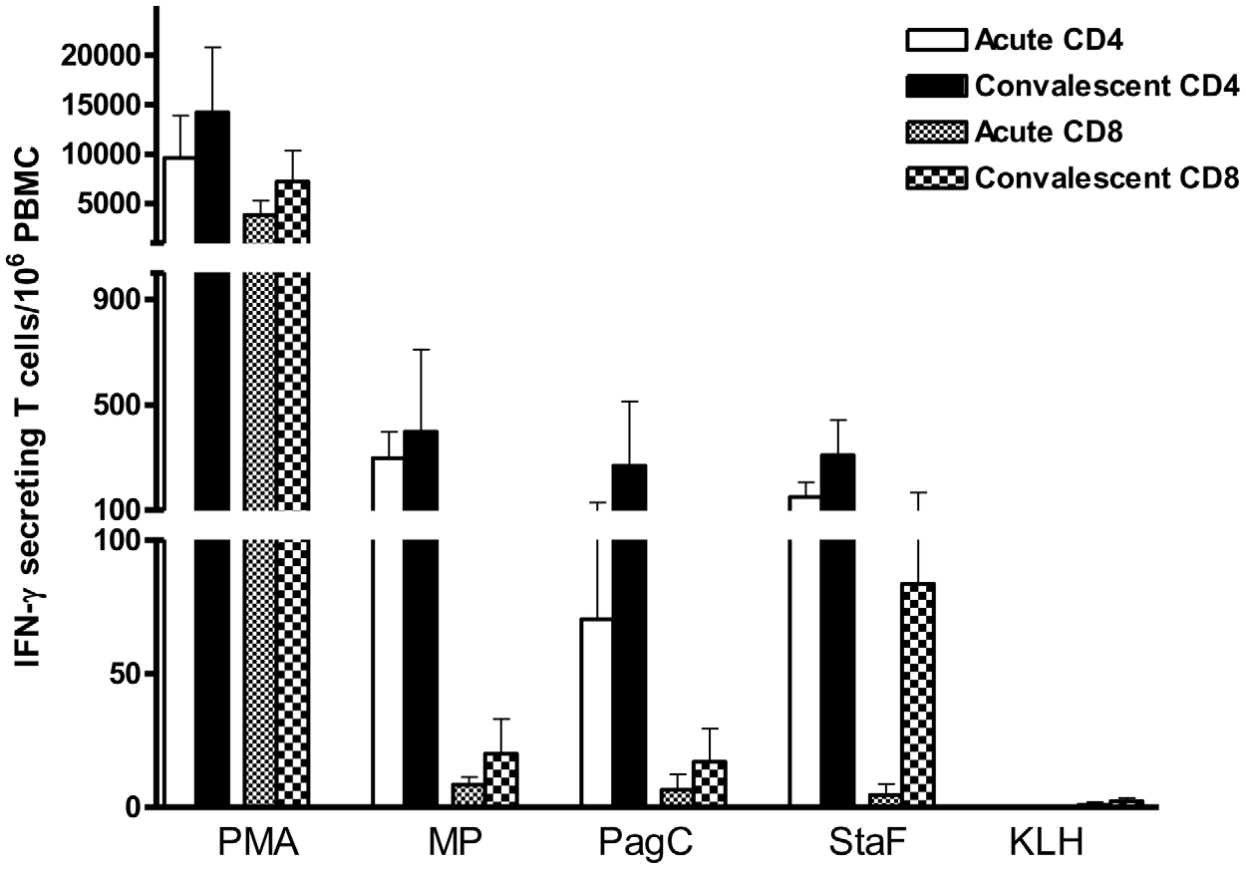
Characterization of interferon-γ CD4 and CD8 responses to *S*. Typhi antigens including StaF (STY0202), PagC (STY1878), *S*. Typhi membrane preparation (MP), and PMA and keyhole limpet hemocyanin (KLH) during acute and convalescent stage illness in *S*. Typhi bacteremic patients. Mean and standard error of the mean represented.

We found that patients with *S*. Typhi bacteremia had elevated interferon-γ ELISPOT responses at both acute and convalescent stages of infection compared to healthy controls for all seven of the purified *S*. Typhi proteins, as well as against *S*. Typhi crude membrane preparation (P<0.05) (Figure 1). In contrast, responses to PHA did not differ significantly between patients and healthy controls, and minimal responses were detected against control protein and KLH in both patients and healthy controls. To assess whether interferon-γ responses were CD4 or CD8-derived, we used intracellular cytokine staining following stimulation with a subset of proteins, and found that the majority of interferon-γ expressing cells were CD4-positive, although a CD8 positive response was also detected (Figure 2).

### Proliferation responses

**Figure 3:**
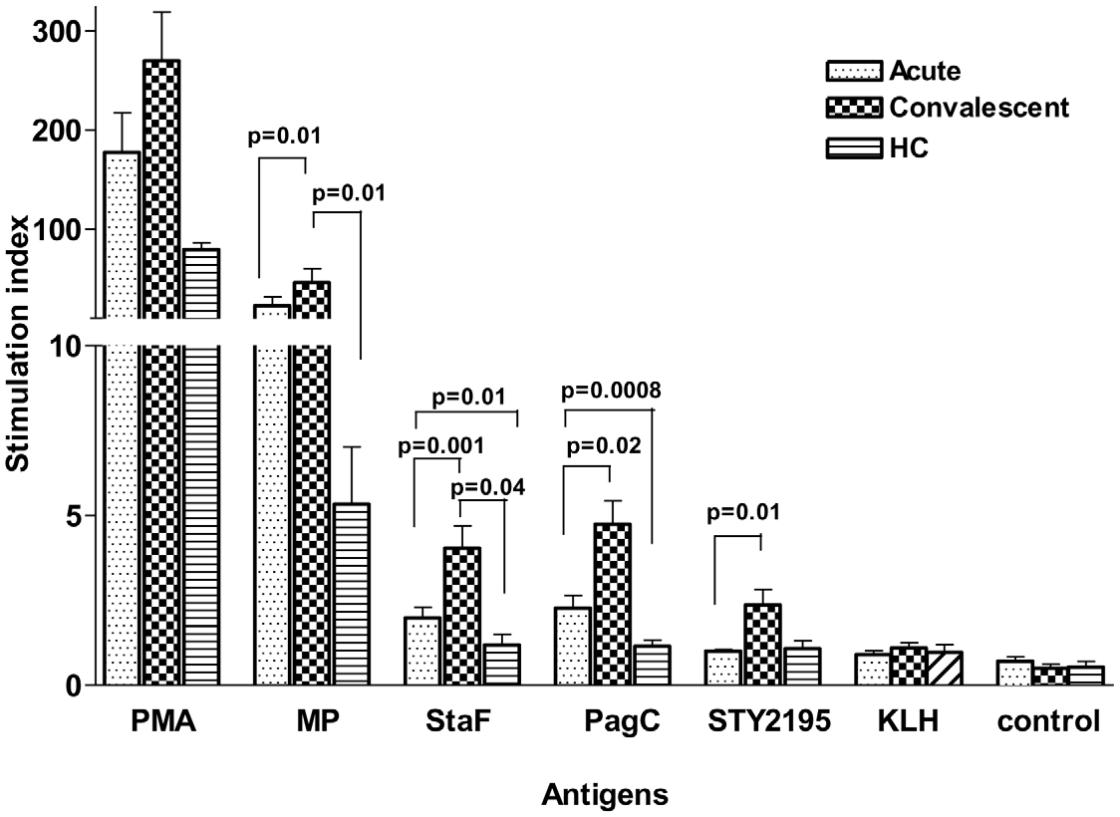
Cellular proliferation responses to *S*. Typhi antigens including StaF (STY0202), PagC (STY1878), conserved hypothetical protein encoded by STY2195, *S*. Typhi membrane preparation (MP), control protein, and phytohaemagglutinin (PHA) and keyhole limpet hemocyanin (KLH) during acute and convalescent stage illness in *S*. Typhi bacteremic patients and in healthy controls (HC). Stimulation index: net cpm with antigen /net cpm without antigen (media alone) for each individual on each day (acute and convalescent). Mean and standard error of the mean represented.

To further evaluate responses, we selected the three proteins associated with the highest interferon-**γ** expression levels in convalescent phase samples, as well as membrane preparation, for inclusion in cellular proliferation assays. In comparison to healthy Bangladeshi controls residing within the same *S*. Typhi endemic area, individuals with documented *S*. Typhi bacteremia had significantly elevated proliferation indices at the acute stage of illness to StaF and PagC (P<0.01−0.0008), but not to STY2195, or crude membrane preparation, and these acute stage responses further significantly increased within bacteremic individuals by the convalescent period compared to the acute stage responses (P≤0.02−0.001) (Figure 3). We also detected a significantly increased proliferation response to STY2195 and *S*. Typhi membrane preparation in bacteremic patients at convalescence compared to acute phase samples and compared to control patients (p≤0.01).

## Discussion

Cellular immune responses, including CD4 and CD8-mediated interferon-γ responses, play a critical role in clearing and controlling systemic *Salmonella* infections [23], [40]. Despite this, there has been limited evaluation of cellular responses in humans to wild-type *S*. Typhi. No animal model fully replicates host-pathogen interactions and immunologic events that occur during this human-restricted infection. Evaluation in humans has largely focused on characterizing responses in recipients of attenuated vaccine strains of *S*. Typhi [23], [38], [41], [42], [43], [44]. We report here a screening approach that permitted us to evaluate interferon-γ and proliferation responses to a number of bacterial antigens in *S*. Typhi-infected humans in Bangladesh. We selected proteins that we had previously identified in immuno-affinity screening assays for humoral responses [28], and we recovered these selected proteins using an automated system and high throughput genomic and proteomic technologies. Although we were able to generate adequate samples for only approximately a third of our selected proteins for evaluation in humans, we feel that high throughput approaches such as the one we describe will assist in accelerating analysis of pathogens that express thousands of antigens. For instance, *S*. Typhi contains approximately 4,400 open reading frames, and although protein microarrays can be used to screen for humoral responses across the immunoproteome, no comparable system has yet been developed to assess cellular immune responses in a high throughput manner, despite the critical role that cellular immune responses play against intracellular pathogens.

We recognize that high throughput purification techniques may be compromised by issues of contamination, including with LPS when expression occurs in *E. coli* vectors. However, LPS contamination of all preparations was found to be less than the level of detection of our assay kit (<300 fg/µl), we did not detect cellular immune responses to control protein expressed and purified from *E. coli* in the same manner as our *S*. Typhi proteins, and we detected cellular immune responses in patients but not healthy controls to purified *S*. Typhi proteins. All of these observations suggest that the responses we observed were antigen-specific and not due to contaminating LPS.

In the *S*. Typhimurium mouse model, CD4 and CD8 cells are critical to the development of protective immunity, and control of *Salmonella* infection involves prominent expression of interferon-γ by both CD4 and CD8 cells [24], [25], [26]. Overall, only a relatively few defined class I and class II epitopes have been identified in the *S*. Typhimurium mouse model, including epitopes in FliC and SipC for CD4 cells, and OmpC and GroEL for CD8 cells [24], [40], [45], [46], [47], [48]. A number of *Salmonella* antigens are also able to induce partially protective immunity when included in subunit-based vaccines in mice, including flagellin, MIG-14 and SseB (*Salmonella* antigens expressed in vivo), suggesting that immune responses against a number of *Salmonella* antigens could contribute to protective immunity [49], [50], [51].

In comparison to the murine data, evaluation of cellular responses to *S*. Typhi in humans have largely involved individuals who have received attenuated *S*. Typhi vaccine strains such as Ty21a and CVD908 [23], [38], [41], [42], [43], [44]. In concordance with the mouse data, these studies have shown induction of interferon-γ-expressing CD4 and CD8 responses following vaccination [23], [24], [38], [42], [52]. Interestingly, CD8 responses may involve both classical (HLA-A, B and C in humans) and non-classical (HLA–E, F, and G) mediated T cell recognition [43], [52]. Using an ex vivo model, Sztein and colleagues have also recently found that direct infection of antigen-presenting dendritic cells with *S*. Typhi leads to expression of high levels of TNF-α, IL-6 and IL-8, and low levels of interferon-γ and IL-12 p70, but that dendritic cells can also ingest other infected human cells leading to high level expression of interferon-γ and IL-12 p70, with subsequent induction of a population of CD3+CD8+CD45RA-CD62L- effector/memory T cells in co-cultured lymphocytes [53]. Based on these observations, we used recombinant antigens to assess and characterize interferon-γ and proliferation responses in infected humans in Bangladesh. To establish the feasibility of our approach, we focused our initial efforts on a subset of proteins that we had previously identified as generating humoral immunity and being expressed in vivo during human infection [28]. These included a number involved in fimbrial attachment or adhesion such as StaF, StbB, CsgF, and CsgD, as well as OppA, a conserved hypothetical protein encoded by STY2195, and PagC, an outer membrane protein encoded by STY1878. We previously found that humans infected with *S*. Typhi develop a serum antibody response to PagC and that this response increases at convalescence [28]. Here we furthered this observation and report detection of a parallel cellular response against PagC during human infection, including both interferon-γ and proliferative responses, and show that responses in convalescence were higher than during acute stage illness. Although the role of PagC during human infection is not fully understood, its expression is controlled by the PhoP-regulon involved in intra-macrophage survival [19], [54].

We also detected significant increases in cellular responses during convalescence against StaF, a fimbrial protein homologous to *E. coli* YadK that contains a Pfam motif believed to be involved in cellular adhesion [17], STY2195, a conserved hypothetical protein of unknown function, and a crude membrane preparation containing over 900 *S*. Typhi proteins, including GroEL, OmpC, OppA and PagC.

We found that *S*. Typhi proteins elicit both CD4^+^ and CD8^+^ interferon-γ expressing responses, with CD4 responses being more numerous than CD8 responses. Of interest, we were able to detect antigen-specific interferon-γ responses in patients, including at the time that patients presented for clinical care, but similar responses were not seen on controls. These observations suggest that an antigen-specific interferon-γ-based detection system might be used to diagnose individuals with typhoid fever during the acute stage of illness, similar to the approach used to diagnose infection with *Mycobacterium tuberculosis* [55], [56], [57]. Currently, all available diagnostic tests for typhoid fever lack either sensitivity and/or specificity, especially in areas of the world endemic for typhoid. For example, microbiological culturing of blood has approximately 30–70% sensitivity, depending on the volume of blood obtained and whether previous antibiotics have been administered, and the Widal assay has at best 85% specificity when analyzing both acute and convalescent phase responses in endemic zones where typhoid exacts its highest burden [58], [59], [60].

In summary, we have used a screening format to preliminarily characterize *S*. Typhi antigen-specific interferon-γ responses in patients with typhoid fever. This is the first characterization of such responses in humans, and further immunologic analysis will be required to assess the role, if any, that these responses play in controlling or clearing *S*. Typhi infection. Our study has a number limitations, including analysis of a relatively small number of purified *S*. Typhi antigens, characterization of a limited number of immunologic parameters, and the absence of the inclusion of febrile control patients confirmed not to be acutely infected with *S*. Typhi; however, our detection of antigen-specific interferon-γ responses could assist in the development of interferon-γ-based diagnostic assays for typhoid fever, and our overall approach could be used to identify antigens capable of inducing cellular immune responses during infection with other intracellular pathogens.

## Supporting Information

Table S1 **Mass spectrometric analysis of** ***S*** **. Typhi membrane preparation.**
(XLS)
